# Batch effects account for the main findings of an in utero human intestinal bacterial colonization study

**DOI:** 10.1186/s40168-020-00949-z

**Published:** 2021-01-12

**Authors:** Marcus C. de Goffau, D. Stephen Charnock-Jones, Gordon C. S. Smith, Julian Parkhill

**Affiliations:** 1 Department of Veterinary Medicine, University of Cambridge, Cambridge, UK; 2 Department of Obstetrics and Gynaecology, National Institute for Health Research Biomedical Research Centre, University of Cambridge, Cambridge, UK; 3 Centre for Trophoblast Research (CTR), Department of Physiology, Development and Neuroscience, University of Cambridge, Cambridge, UK

**Keywords:** Batch effects, Decontam, Colonization in utero, 16S rRNA

## Abstract

A recent study by Rackaityte et al. reported evidence for a low level of bacterial colonization, specifically of *Micrococcus luteus*, in the intestine of second trimester human fetuses. We have re-analyzed their sequence data and identified a batch effect which violates the underlying assumptions of the bioinformatic method used for contamination removal. This batch effect resulted in *Micrococcus* not being identified as a contaminant in the original work and being falsely assigned to the fetal samples. We further provide evidence that the micrographs presented by Rackaityte et al. are unlikely to show Micrococci or other bacteria as the size of the particles shown exceeds that of related bacterial cells. Finally, phylogenetic analysis showed that the microbes cultured from the fetal samples differed significantly from those detected by sequencing. Overall, our findings show that the presence of *Micrococcus* in the fetal gut is not supported by the primary sequence data. Our findings underline important aspects of the nature of contamination for both sequencing and culture approaches in microbiome studies and the appropriate use of automated contamination identification tools.

## Main text

A recent study by Rackaityte et al. [1] reported evidence for a low level of bacterial colonization of the fetal intestine from second trimester human fetuses. The authors reported V4 16S rRNA gene amplification sequence data from both meconium samples and various negative controls, including several types of swabs and fetal kidney samples. They used the R package decontam [2] to account for reagent contamination and, after filtering, found several signals of potential interest that appeared to be enriched in fetal meconium compared to their controls. Quantitative PCR, fluorescent in situ hybridization (FISH), scanning electron microscopy (SEM), phenotypic characterization of lamina propria T cells, RNAseq of fetal intestinal epithelial cells and culture were performed and appeared to support the presence of microbes, possibly including *Micrococcus luteus*. Our re-analysis of the data however provides strong evidence that several of the findings are caused by an unrecognized batch effect.

## Batch effect in 16S rRNA gene amplicon sequencing data

**Fig. 1:**
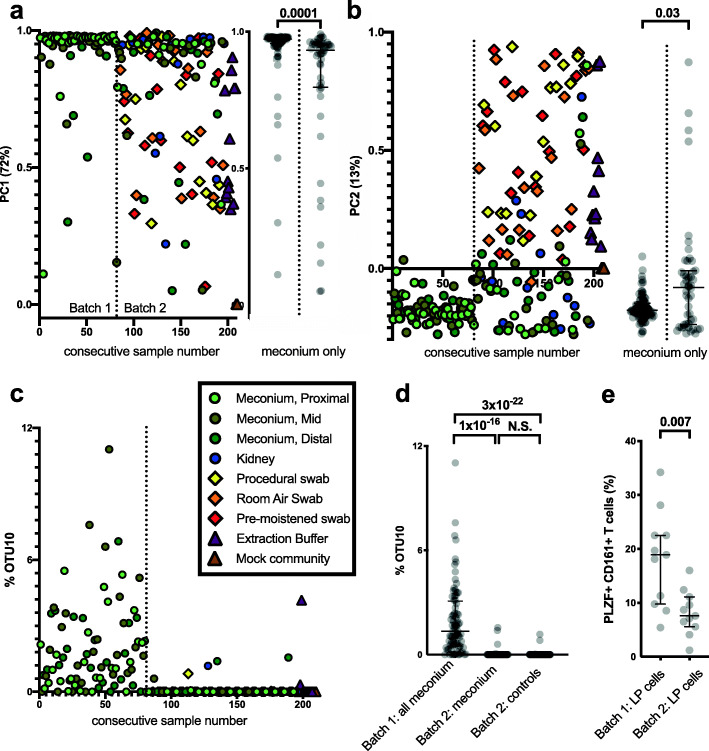
Batch effect analysis of V4 16S rRNA gene amplification data with a focus on *Micrococcus* (OTU10). Source data: Supplemental Table 2 of Rackaityte et al. [2]. **a**, **b** Principal components 1 and 2 (PC1 and PC2), respectively, show a distinct microbial profile shift after sample 80 (ID 1616 L), as indicated with a dashed vertical line and designated as batch 1 and batch 2 as indicated in **a**. A sub-analysis of PC1 and of PC2 with the meconium samples only is shown to the right of both figures. **c** The *Micrococcus* signal (OTU10) is part of this profile shift. **d** *Micrococcus* (OTU10) signals from batch 1 (samples 1–80 all meconium) vs meconium only samples from batch 2 or vs all negative controls from batch 2 show that the OTU10 signal is batch associated. **e** The phenotypic characterization of the lamina propria (LP) T cells shows that samples corresponding to batch 1 had significantly higher proportions of PLZF^+^CD161^+^T cells compared to samples corresponding to batch 2. Interquartile ranges are shown, and comparisons indicated by brackets have *P* values shown above them (Mann-Whitney *U* test)

We reanalyzed their V4 16S rRNA gene amplification data using metadata reported in Supplemental Table 2 (the unfiltered OTU table) excluding samples with fewer than 100 reads. Read numbers for each OTU were normalized into a percentage of the total number of reads per sample. Principal component analysis (PCA) was performed to identify whether the main sources of variation in the data were associated with the sample type or were due to sample-independent (batch) effects. Interestingly, PC1 (72%), PC2 (13%) (Fig. 1a, b), and PC3 (4%) demonstrated that the first 80 samples (as ordered by the authors’ identifier) appeared to have a different microbial profile to the next 130, irrespective of the actual source of the sample. This analysis suggests that some aspect of the sample collection or processing was performed in at least two batches. The authors state in their methods, and have confirmed to us, that all the sequencing was performed in a single batch. However, it is apparent that there was some change in their technical procedures, for example, a change in sample collection procedures coinciding with a switch from sampling meconium only to sampling meconium and additional controls, or a different lot of one or more collection reagents used during the period over which the samples were collected. Importantly, this switch coincides with a clear change in the microbial profile. Before the switch, samples were solely composed of meconium (from 28 fetal donors) and had been taken from the proximal, mid, and distal sections of the small intestine (ID numbers 1519–1616, D, J, and L, respectively) and included no controls. After the switch, the samples included meconium from 22 fetal donors (three sites each) and four negative controls for 19 of the fetal samples (ID numbers 1633–1660): a procedural swab, a room air swab, a moistened swab, and a kidney sample (S, A, N, and K, respectively). The remainder of the samples comprised 11 extraction buffer samples and 3 mock community samples, and these clustered with the second batch by our PCA analysis.

The R package decontam [2] employed by Rackaityte et al. to differentiate between real and contaminant signals depends on the differential presence of contaminants between real samples and negative controls and therefore requires that the different sample types are collected and processed contemporaneously and with identical reagent lots. We and others have shown that contamination can occur at multiple points in the procedure, including during sample collection, sample washing, DNA extraction, DNA amplification, and sequencing [3–8]. Moreover, variation in contamination is introduced at each step in the process due to using different batches or lot numbers of buffers, DNA extraction kits, mtDNA removal, or PCR reagents—all of which vary in the amount and type of contamination. Hence, the decontam R package cannot be used in the presence of an obvious batch effect—whatever the source—when the negative controls are differentially distributed across the batches. The nature of the different batches is unlikely to be known and unless all steps are carried out in a single batch then all the samples need to be randomized. In any event, the data should be examined to specifically identify possible batch effects.

**Fig. 2:**
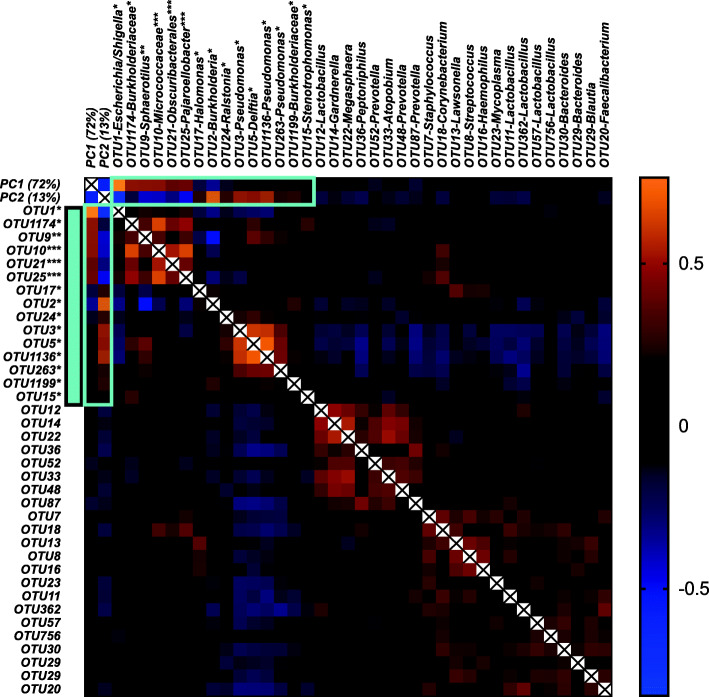
Spearman rho correlation analysis of PC1 and PC2 with the most abundant or relevant OTUs. The color of each block in the heatmap indicates the strength of correlation between each OTU and PC1 or PC2, or with other OTUs. The 3 mock community samples were excluded. Likely, contaminants show strong correlations with PC1 and PC2, and with each other, and are indicated with the light blue vertical bar on the left. OTUs indicated with a single asterisk were identified by decontam as a contaminant and were removed after filtering. Double asterisk indicates that they were not identified by decontam but that they were removed after filtering, and triple asterisk indicates OTUs that were not identified by decontam and were also not removed after filtering. The light blue boxes within the heatmap indicate strong correlation with PC1 and/or PC2 (the batch effect) and/or that they were strongly correlated with one another and abundantly present in negative controls

We performed a systematic analysis to determine which specific OTUs initially assigned to fetal samples were affected by the batch effect (Fig. 2). This analysis demonstrated that not only “*Micrococcus*” (OTU10) but also OTU21 (Obscuribacterales) and OTU25 (*Pajaroellobacter*) are strongly associated with PC1 and PC2 and are therefore likely contaminants. In this case, we believe that *Micrococcus* (OTU10) was not recognized as a contaminant because controls were only included in batch 2. Two comparisons demonstrate that *Micrococcus* (OTU10), and also others such as OTU21 (Obscuribacterales) and OTU25 (*Pajaroellobacter*), are likely contaminants. First, *Micrococcus* (OTU10) is detected mainly in batch 1, which only consists of meconium samples and no negative controls and is mostly absent in batch 2 (Fig. 1c). When comparing batch 1 meconium samples with batch 2 meconium samples (Fig. 1d), a significant difference in its prevalence is observed (*P* = 1 × 10^− 16^) demonstrating that the presence of OTU10 in meconium samples is not associated with the sample type but with the batch. Second, and crucially, when considering only batch 2 (which included both samples and controls), there is no difference between the presence of *Micrococcus* (OTU10) in the meconium samples and the controls (*P* = 0.9). The same is true for OTU21 and OTU25. It is therefore likely that OTU10 (or its DNA) is indeed present in the first set of samples. However, it is very unlikely to be from the meconium itself, as it is absent in the second set of meconium samples (and the associated controls).

## Batch effect in lamina propria T cell data

Interestingly, this batch effect was also found in the phenotypic characterization of lamina propria T cells (Fig. 1e) as samples from batch 1 had significantly higher proportions of PLZF^+^CD161^+^T cells compared to samples corresponding to batch 2 (*P* = 0.007). In addition, the samples used for the RNAseq analysis were selected as being *Micrococcaceae*-meconium associated epithelium (MM-E, *n* = 7), *Lactobacillus*-meconium associated epithelium (ML-E, *n* = 3), and other-meconium epithelium (OM-E, *n* = 3). However, these samples also segregate by batch with all the MM-E samples and none of the LM-E or OM-E coming from batch 1. There are two potential explanations for the observations on both the microbiota profile (Fig. 1a–d) and the phenotypic characterization of lamina propria T cells (Fig. 1e): (1) a change in collection procedures, cell isolation, or analysis reagents was associated with the switch from sampling meconium only to sampling meconium and controls and this affected the host tissues taken at the same time, leading to these different results or (2) there is an undescribed biological difference between the first and the second batch of samples. This explanation seems unlikely as the samples represent a random collection, as confirmed to us by the authors. However, if this was the case, a significant unaccounted biological difference between the samples that were collected with controls, and those that were collected without, would invalidate the subsequent analyses.

## Microscopy analysis

**Fig. 3:**
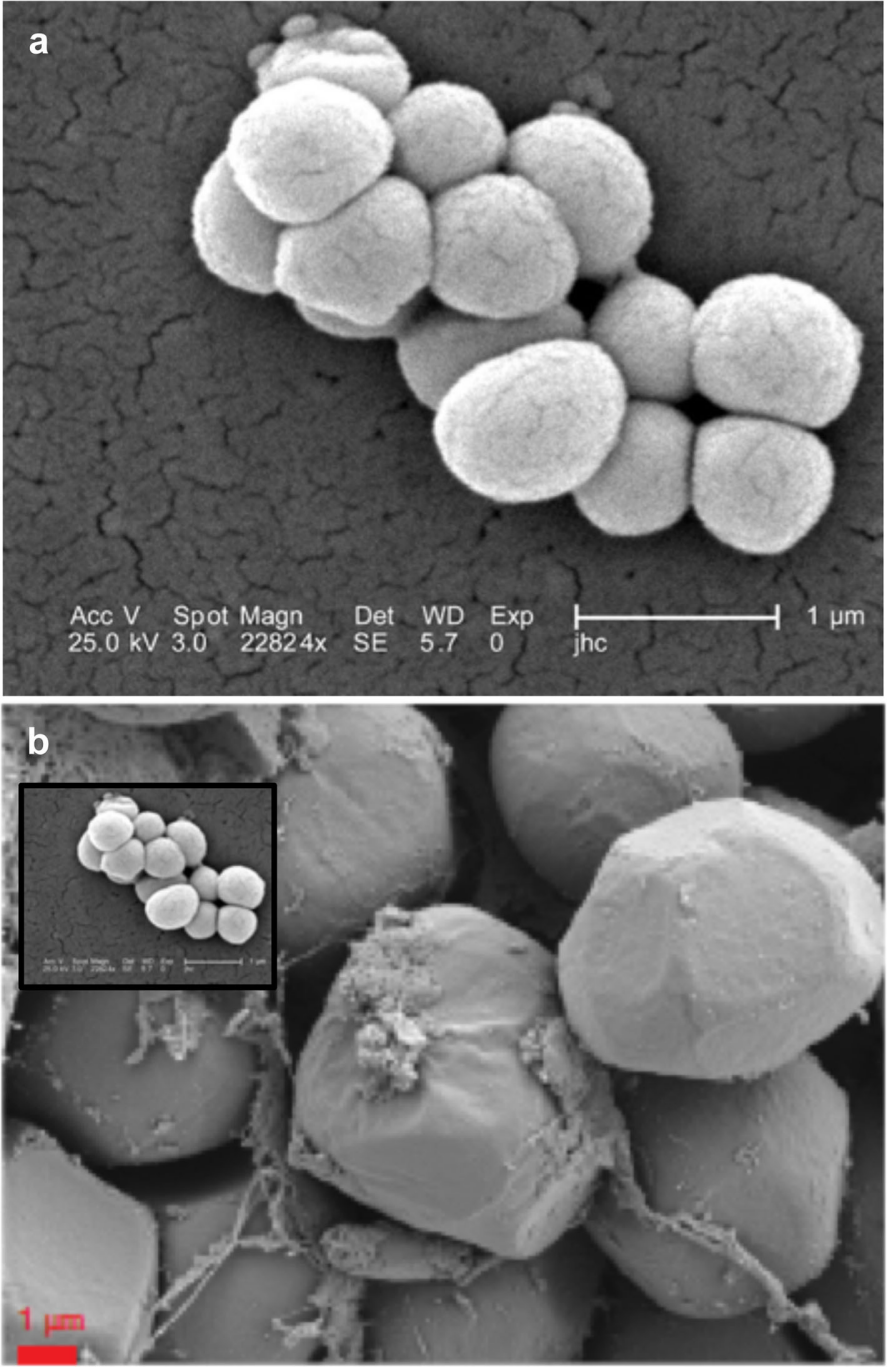
Scanning electron micrograph of **a** *Micrococcus luteus*, source: CDC, identification number 9759, photo credit: Janice Carr. https://commons.wikimedia.org/wiki/File:Micrococcus_luteus_9759.jpeg, and **b** Fig. 1b (right panel) of Rackaityte et al. Inset Fig. 2a drawn to the same scale as Fig. 2b to provide a direct comparison of both pictures (based on length of 1 μm scale bars)

The SEM images shown in Fig. 1 by Rackaityte et al. [1] show coccus-like structures that are much larger than *M. luteus* or other bacterial species reported in the paper. The diameter of *M. luteus* is typically 0.4–2.2 μm (Fig. 3), depending on their metabolic state [9]. The coccoid structures reported by Rackaityte et al. are 4–5 μm (an average measured from all the panels in Fig. 1 in that paper) [1]. It is not apparent what the structures visualized by SEM actually represent, as the morphology of the coccus-like particles does not resemble the morphology of cocci or other bacteria discussed in the paper. Demonstrating that they are specific bacteria would require the use of species-specific fluorescent in situ hybridization (FISH) probes and sufficient magnification such that the morphology of individual cells can be analyzed in detail. Unfortunately, in this report, only species-non-specific FISH probes have been used, and they are presented at an insufficient magnification to identify the morphology of any of the individual cells.

## *Micrococcus luteus* culture and biology

Accepted knowledge of the biology of Micrococci casts doubts on the interpretations of the findings obtained by culturing. *M. luteus* is one of the easiest organisms to culture and is often found to appear on agar plates as yellow colonies even when apparently working aseptically. *M. luteus* is an obligate aerobe; it is found in soil, dust, water, and air, and as part of the normal microbiota of the mammalian skin. *M. luteus* can withstand very high doses of UV radiation [10, 11] and is similarly adept at surviving desiccation and starvation [12, 13]. Accidental culture of contaminants can occur in even the most rigorously controlled environments [14]. Significant efforts were made to culture this organism, which initially could not be cultured on standard medium. If this was due to these *M. luteus* cells being dormant in utero (as suggested), their size would have been 0.4 μm [9] and they would have only required resuscitation promoting factors (RpFs) for growth, as is well described in the literature [15]. Attempting to resuscitate *M. luteus* by adding host factors from an environment where *M. luteus* had supposedly become dormant seems at odds with common knowledge on the biology of Micrococci. It is at least plausible that the culture of *M. luteus* was due to environmental contamination.

## Sequence comparison of OTU10 with cultured *Micrococcus luteus*

**Fig. 4:**
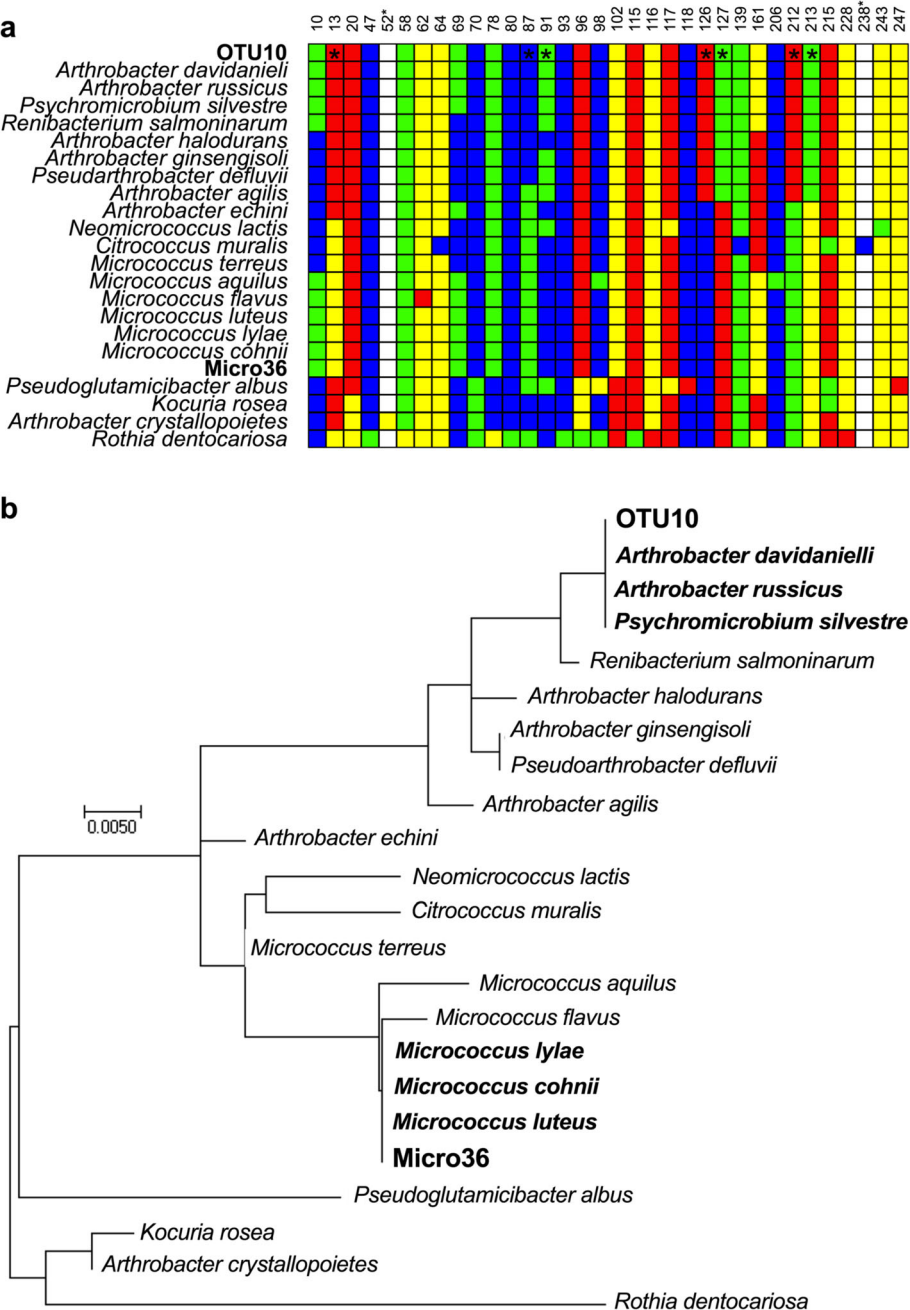
Taxonomic analysis of the 16S rRNA V4 region of Micro36 and OTU10. **a** Comparison between the 16S rRNA V4 region sequence of Micro36, OTU10, and various other *Micrococcaceae*. Variable nucleotides in the alignment are shown in green (A), red (T), blue (C), and yellow (G). White indicates an insertion in one of the other *Micrococcaceae*. The numbering is that used in Extended Data Fig. 5 of Rackaityte et al. [1]. Nucleotides dissimilar between Micro36 and OTU10 are highlighted with an asterisk. **b** A phylogeny inferred from the 16S rRNA V4 region sequences using the maximum likelihood method based on the Tamura-Nei model [17]. The tree is drawn to scale, with branch lengths representing the number of substitutions per site. Evolutionary analyses were conducted in MEGA7

Finally, the 16S rRNA V4 region of the cultured *M. luteus* (Micro36) and OTU10 differ by seven nucleotides as shown in Extended Data Fig. 5 [1]. We analyzed the sequences of both Micro36 and of OTU10 initially with Blastn [16] (NCBI online BLAST interface (blastn, Standard databases, exclude uncultured, http://blast.ncbi.nlm.nih.gov). The 16S rRNA V4 region of Micro36 had 100% similarity with *Micrococcus luteus* and various other *Micrococcus* species. However, OTU10 had 100% similarity with *Arthrobacter davidanieli*, *A. russicus*, and *Psychromicrobium silvestre*. These three species are *Micrococcaceae* but are not coccoid; they are in fact coryneform soil bacteria. A phylogeny built using the 16S rRNA V4 region of Micro36 and OTU10 and several other *Micrococcaceae* (Fig. 4) shows that they are clearly distinct. At a SNP level, these three species do not share a single one of the 7 single nucleotide polymorphisms (SNPs) with any of the *Micrococcus* species, but do share many of the same SNPs with various other *Arthrobacter* species or species/genera closely related to *Arthrobacter*. It is therefore apparent that neither the strain the authors have cultured nor the structures visible in their SEMs actually correspond to the OTU10 identified in their 16S analysis.

## Conclusion

These data indicate that the most likely explanation for the identification of *Micrococcus* in these fetal gut samples is contamination. Most importantly, and most relevant in a wider scientific context, the 16S rRNA sequencing data is unreliable due to unequal distribution of negative controls between batches. Batch effects matter. Even running an effective decontamination program, like decontam, will not be sufficient if the underlying assumptions of the software have been violated by the experimental design. As discussed in the paper reporting decontam [2], it uses statistical methods to identify contaminant sequences in metagenomic data based on two widely reproduced patterns: contaminants appear at higher frequencies in low-concentration samples and are found more frequently in negative controls than in samples. If there is a batch effect and an unequal distribution of negative controls among the batches, such as with the study of Rackaityte et al., it cannot remove contaminants that are specific to particular batches with insufficient (or zero) negative controls, even if it is stringently implemented. Decontam is an excellent tool for helping improve the quality of metagenomic and marker gene sequencing studies, but its requirements need to be respected. Other methods of identifying contamination exist and we strongly recommend using multiple approaches (including decontam) as contamination in low-biomass studies is pernicious and pervasive, and there are multiple sources of contamination [3].

## Data Availability

All data used within this article can be obtained from the published manuscript under discussion (https://www-nature-com.ezp.lib.cam.ac.uk/articles/s41591-020-0761-3).
